# Decreasing Delirium through Music (DDM) in critically ill, mechanically ventilated patients in the intensive care unit: study protocol for a pilot randomized controlled trial

**DOI:** 10.1186/s13063-017-2324-6

**Published:** 2017-11-29

**Authors:** Sikandar H. Khan, Sophia Wang, Amanda Harrawood, Stephanie Martinez, Annie Heiderscheit, Linda Chlan, Anthony J. Perkins, Wanzhu Tu, Malaz Boustani, Babar Khan

**Affiliations:** 1 Division of Pulmonary, Critical Care, Sleep and Occupational Medicine, Department of Medicine, Indiana University School of Medicine, Indianapolis, IN USA; 2 Department of Psychiatry, Indiana University School of Medicine, Indianapolis, IN USA; 3 Indiana University Center of Health Innovation and Implementation Science, Indianapolis, IN USA; 4 IU Center of Aging Research, Regenstrief Institute, Indianapolis, IN USA; 5 Music Therapy Department, Augsburg College, Minneapolis, MN USA; 6 Department of Nursing, Nursing Research Division, Mayo Clinic, Rochester, MN USA; 7 Indiana Clinical and Translational Research Institute, Indiana University School of Medicine, Indianapolis, IN USA; 8 Department of Biostatistics, Indiana University School of Medicine, Indianapolis, IN USA; 9 Sandra Eskenazi Center for Brain Care Innovation, Eskenazi Hospital, Indianapolis, IN USA; 10 Division of Geriatrics and General Internal Medicine, Department of Internal Medicine, Indiana University School of Medicine, Indianapolis, IN USA

**Keywords:** Delirium, Music, Critical care

## Abstract

**Background:**

Delirium is a highly prevalent and morbid syndrome in intensive care units (ICUs). Changing the stressful environment within the ICU via music may be an effective and a scalable way to reduce the burden of delirium.

**Methods/design:**

The Decreasing Delirium through Music (DDM) study is a three-arm, single-blind, randomized controlled feasibility trial.

Sixty patients admitted to the ICU with respiratory failure requiring mechanical ventilation will be randomized to one of three arms (20 participants per arm): (1) personalized music, (2) non-personalized relaxing music, or (3) attention-control. Music preferences will be obtained from all enrolled participants or their family caregivers. Participants will receive two 1-h audio sessions a day through noise-cancelling headphones and mp3 players. Our primary aim is to determine the feasibility of the trial design (recruitment, adherence, participant retention, design and delivery of the music intervention). Our secondary aim is to estimate the potential effect size of patient-preferred music listening in reducing delirium, as measured by the Confusion Assessment Method for the ICU (CAM-ICU). Participants will receive twice daily assessments for level of sedation and presence of delirium. Enrolled participants will be followed in the hospital until death, discharge, or up to 28 days, and seen in the Critical Care Recovery Clinic at 90 days.

**Discussion:**

DDM is a feasibility trial to provide personalized and non-personalized music interventions for critically ill, mechanically ventilated patients. Our trial will also estimate the preliminary efficacy of music interventions on reducing delirium incidence and severity.

**Trial registration:**

ClinicalTrials.gov, Identifier: NCT03095443. Registered on 23 March 2017.

**Electronic supplementary material:**

The online version of this article (doi:10.1186/s13063-017-2324-6) contains supplementary material, which is available to authorized users.

## Background

Delirium is characterized by a disturbance of consciousness, the presence of inattention, disorganized thinking, and a fluctuating course [1]. It has an incidence as high as 85% in mechanically ventilated patients, and is associated with longer hospital stays, increased hospital and post-discharge mortality as well as higher healthcare costs [2–6]. Furthermore, over 30% of patients with delirium experience long-term cognitive impairment [7].

Despite the incidence and morbidity associated with delirium, effective pharmacological and non-pharmacological interventions to treat this condition are lacking. Music listening is a unique, non-pharmacological intervention that may reduce delirium incidence and severity through the inhibition of inflammation. Prior music listening research has demonstrated a calming effect with slow-tempo music. This is hypothesized to be secondary to entrainment of the autonomic nervous system, reduction in sympathetic activation and dampening of the inflammatory state [8]. With various pathophysiological models for delirium implicating neuro-inflammation, neurotransmitter imbalances and an aberrant stress reaction, [9] this makes music listening a promising intervention to prevent or treat delirium.

Music intervention studies in the intensive care unit (ICU) have focused on its effects on anxiety, pain, tolerance of non-invasive ventilation, and physiological indicators of the stress response (heart rate, blood pressure, catecholamines, cortisol), but, to date, studies on the effect of music listening on ICU-related delirium have not been conducted [10–16]. Our study aims to test the feasibility of music listening, and estimate the potential effect size of music listening on delirium, in critically ill patients on mechanical ventilation.

## Methods/design

### Rationale

We are conducting a feasibility trial to deliver personalized music for critically ill patients based on preferences supplied by their surrogates or family members. Participants in the study are unlikely to be able to communicate or choose their own music due to invasive mechanical ventilation, the need for high ventilator support, and hemodynamic instability. Our study design also includes single blinding and three intervention arms (personalized music, non-personalized, investigator-selected music, and attention-control) which adds complexity to the unique ICU environment.

Our study aims to gather data to help inform a definitive randomized controlled trial.

### Outcomes

Our study aims to answer the following question: In critically ill patients on mechanical ventilation, is the design and implementation of music listening feasible?

The primary outcomes of our study are: (1) to assess recruitment rates, described by the number of participants approached and their rates of consent, (2) to assess adherence to the intervention by number of listening sessions completed per patient, and (3) to assess retention by the number of participants completing the study.

The secondary outcome in the study is to estimate the potential effect size of patient-preferred music in reducing delirium incidence as measured by the Confusion Assessment Method for the ICU (CAM-ICU).

### Design

DDM is a feasibility study with a three-arm, single-blind, randomized controlled design.

**Fig. 1:**
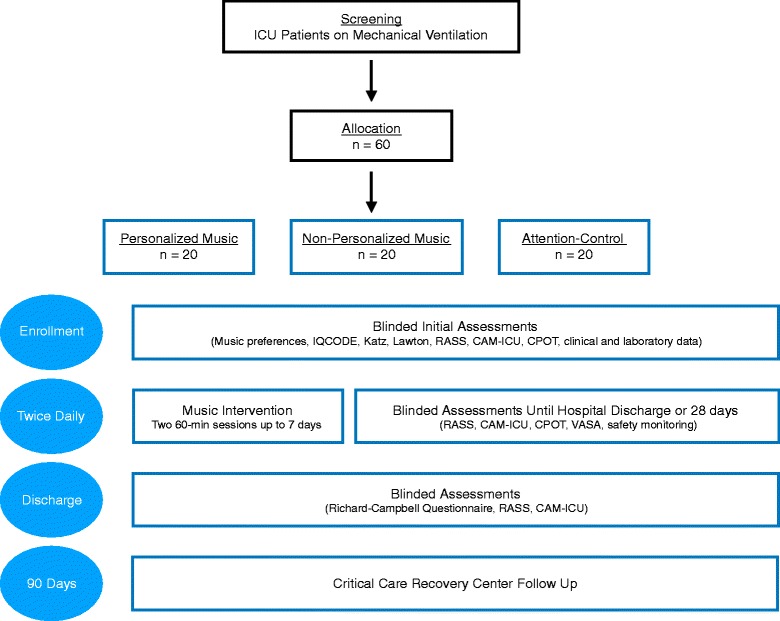
DDM study flowchart, interventions, and assessments. Abbreviations: *CAM-ICU* Confusion Assessment Method for the Intensive Care Unit, *CPOT* Critical Pain Observation Tool, *Katz* Katz Index of Independence in Activities of Daily Living, *Lawton* Lawton Instrumental Activities of Daily Living, *RASS* Richmond Agitation-Sedation Scale, *VASA* Visual Analog Scale for Anxiety

Patients admitted to the ICU with respiratory failure requiring mechanical ventilation are eligible. Sixty participants will be randomized to one of three arms (20 patients per arm): (1) personalized music, (2) non-personalized relaxing music, or (3) attention-control (see Fig. 1). Participants will receive two 1-h audio sessions a day through noise-cancelling headphones (and mp3 players). We chose an attention-control arm (rather than usual care) to test the feasibility of a placebo, and to control for the non-specific effects of headphone and non-audio sounds in the study.

**Fig. 2:**
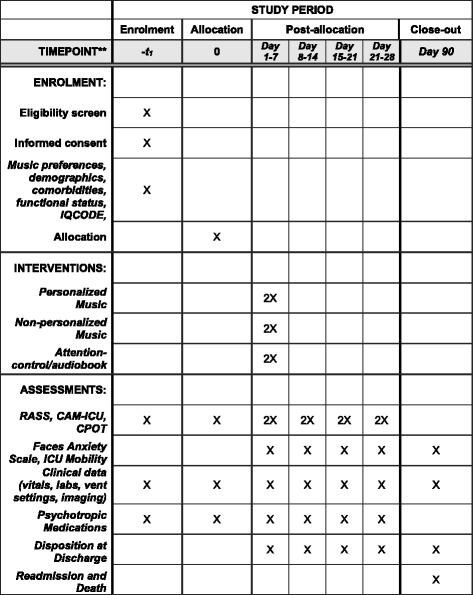
Standard Protocol Items: Recommendations for Interventional Trials (SPIRIT) Figure for the DDM Trial. Abbreviations: *CAM-ICU* Confusion Assessment Method for the Intensive Care Unit, *CPOT* Critical Pain Observation Tool, *ICU* intensive care unit, *IQCODE* Informant Questionnaire on Cognitive Decline in the Elderly, *RASS* Richmond Agitation-Sedation Scale

The trial protocol was written according to the Standard Protocol Items: Recommendations for Interventional Trials Statement (SPIRIT). A SPIRIT Figure is provided (see Fig. 2) and a SPIRIT Checklist is included as Additional file 1.

### Setting

Patients admitted to the medical or surgical ICU service at Sidney and Lois Eskenazi Hospital (Indianapolis, Indiana) are eligible. Eskenazi Hospital is a busy 618-bed, urban, safety net hospital with 44 ICU beds (22 surgical, 22 medical), averaging 120 ICU admissions per month. Demographics of this ICU are: mean age of patients 53.7 years, 45% African American, 47% female, 29% age 60 years or older, and 16% with Medicaid insurance.

### Participants

The target population for our trial is adults aged 18 years and older, admitted to the medical or surgical ICU on invasive mechanical ventilation.

### Consent

Individuals deemed eligible for the study will be approached for consent. If on mechanical ventilation and unable to consent, the patient’s surrogate or healthcare representative will be approached for consent.

### Inclusion criteria

Inclusion criteria for our study are: (1) age 18 years or older, (2) English speaking, (3) admitted to the ICU (medical or surgical), and (4) receiving mechanical ventilation for an anticipated duration of 24 h or longer, provided that the patient has not already been on the ventilator longer than 48 h at the time of enrollment.

### Exclusion criteria

Patients are excluded from our study if any of the following conditions are present: (1) severe dementia or chronic neurological disease, with an IQCODE score of 4.5 or greater; [17–19] (2) acute or subacute severe neurological deficit or injury; (3) severe psychiatric illness (not including depression) or developmental problems; (4) suspected or confirmed drug or alcohol intoxication/overdose or withdrawal; (4) uncorrected or severe hearing or vision impairment; (5) coma after cardiac arrest and/or hypothermia protocol; (6) pregnant or nursing women; (7) prisoners; (8) expected death within 24 h of enrollment or lack of commitment to aggressive treatment by family or the medical team; (9) inability to obtain consent from legally authorized representative within 72 h of meeting eligibility; and (10) attending physician or healthcare team refusal.

### Materials

Participants are provided with noise-cancelling headphones (Maxell NC-IV Superior Noise Cancellation), with mp3 player devices (Apple iPod Shuffle 2GB, 6th generation or Goldenseller 16GB Mp3 Player) during the trial. Music tracks and audiobooks were purchased from Apple Music Store (iTunes).

## Study procedures

### Screening

Patients admitted to the ICU are screened on a daily basis. Music preference data will be collected from all enrolled participants (through their surrogates) regardless of intervention arm.

### Participant randomization

Using a computer-generated list of random numbers created by study statistician, participants are randomized by permuted block with varying block sizes. Randomization occurs within 48 h of enrollment in a 1:1:1 ratio, and only the unblinded study coordinator, who also programs the music devices, is aware of the randomization assignment.

### Study duration/termination

The music intervention period is 7 days post randomization. Participants will stop receiving music sessions early if they choose to withdraw, are transferred out of the ICU, die, or are discharged. In-hospital follow-up continues to 28 days or until discharge, whichever occurs earlier, by daily assessment, unless they choose to withdraw. All participants will be invited for follow-up in the Critical Care Recovery Clinic at 90 days.

### Pre-intervention phase

Upon enrollment, demographics, clinical data (vital signs, pertinent laboratory and imaging data), severity of illness (APACHE II and SOFA), comorbidity burden (Charlson Comorbidity Index), pertinent home medications, reason for admission, pre-hospital functional status (Katz and Lawton), and cognitive function (Informant Questionnaire on Cognitive Decline in the Elderly (IQCODE)) are obtained [20–23]. Cumulative doses of antipsychotic, analgesic, sedative, and anxiolytic medications are also recorded.

A previously described music-preference assessment tool is utilized to obtain the participants’ music preferences from family members/surrogates [24]. We collect information about genre likes and dislikes, as well as specific favorite artists, tracks, and instruments.

A pre-intervention level of consciousness and delirium assessment is performed upon enrollment through the Richmond Agitation Sedation Scale and the CAM-ICU, respectively [3, 25].

### Playlist development

Following enrollment consent, blinded research personnel enter the participant’s music preferences into a Research Electronic Data Capture (REDCap) database. The unblinded study coordinator programs music devices based on an individual study participant’s randomization status. For those randomized to personalized music, Apple Music is utilized to create a playlist based on their musical interests.

Participants randomized to non-personalized music received an mp3 player programmed with slow-tempo, relaxing music and instrumental sounds (piano, relaxing rainfall sounds, and classical music). Finally, participants randomized to attention-control receive a device with audiobooks. The audiobooks include *Treasure Island*, *Harry Potter*, and Dr. Seuss’ *Oh the Places You’ll Go!*, and were chosen for their readability and for their broad appeal.

The playlist for each participant is carefully tracked, which will permit subsequent analysis should particular books or tracks be associated with increased delirium incidence or severity.

To avoid unblinding study personnel to the intervention arm, the coordinator waits a minimum of 4 h to release the mp3 device for use in the study. Prior to allocating devices to patient rooms, all devices are checked to ensure that identifying data including name, artist, collection, and genre have been removed, the device is clean and in good functioning order, and volume is set to a pre-specified level.

### Intervention phase

Music listening sessions are twice a day, for 1 h at a time, from 10:00 to 11:00 and 14:00 to 15:00, for 7 days. The times are based on feedback from ICU nurses associated with least interference with clinical activities. Music listening is provided for 7 days unless the participant refuses, withdraws, is transferred out of the ICU, or dies. Participants extubated within the 7-day period continue to receive the intervention unless they are transferred out of the ICU. Participants remaining in the ICU after 7 days will not receive further intervention due to the resource limitations of our feasibility study.

During listening sessions, a conducive listening environment is created; medical teams are asked to avoid non-urgent patient care activities, the in-room television is turned off, family members are asked to limit their interactions with the patient, and the door is closed to limit outside noise. As all three arms of the trial will receive a conducive listening environment, and due to the use of noise-cancelling headphones in these settings, we believe that the audio dose will remain as the pure active intervention.

Participants receive their first dose of music listening, in one of these windows, as soon after enrollment as possible.

The ICU clinical nurses receive on-site training in the study protocol, and in the operation of the music devices and headphones. Blinded research personnel and/or hospital nurses start the music sessions by placing the headphones on the participants and activating the audio. Adherence is monitored by recording the start and stop date and times for each session. The participant’s heart rate and blood pressure pre and post session are also recorded.

All participants in the trial receive the ABCDEF bundle that is standard of care in the ICU where the study is being conducted. The ABCDEF bundled delirium care protocol places a multidisciplinary focus on daily ventilator liberation trials, sedation vacation, choice and optimization of sedation, nursing-led delirium screenings, early mobility, and family/social support [26]. Participants in the study will continue to have physician-determined sedation without input on choice of sedation from our trial.

### Blinding during intervention

We are using various measures to ensure concealment of a participant’s allocation. Only the unblinded study coordinator programs the audio devices, and ensures that the tracks are anonymized (renamed to a series of digits or letters), to prevent unblinding when bedside staff view the audio device’s screen. Our protocol also benefits from the fact that the majority of audio devices lack screens entirely (Apple iPod Shuffle). Participants, their family members/surrogates, and hospital nurses are asked not to share a patient’s randomization with anyone in the study, should they become aware of it. Audio is not turned on until the headphones are securely placed on the participant to prevent study personnel from inadvertently hearing the output. Participants are permitted to change tracks or adjust the volume if needed. Should one of the study personnel become unblinded, they are reassigned to other study participants. To assess the fidelity of these efforts, we will survey research personnel to predict patient randomization in a subset of participants.

### Assessments during intervention

All assessments are performed by blinded research assistants. Assessments occur twice a day, approximately at 11:00 and 15:00 (after audio interventions). Study personnel perform the following: (1) Richmond Agitation-Sedation Scale (RASS) to document level of consciousness; (2) Confusion Assessment Method for the ICU (CAM-ICU) to screen for delirium [3, 25, 27], and CAM-ICU-7 to determine delirium severity [28]; (3) Critical Care Pain Observation Tool (CPOT) to determine level of analgesia and symptom control [29]; (4) Faces Anxiety Scale (performed after the morning listening session) [30, 31]; (5) physical therapy and occupational therapy notes are evaluated and each participant’s mobility milestones are recorded using Hodgson Mobility Scale [32]; and (6) adverse events are assessed (including seizures while listening, localized skin breakdown or cellulitis around the ears) and are reported to the Institutional Review Board (IRB) per usual protocol.

### Post intervention

Participants receive in-hospital assessments by blinded research personnel until hospital discharge, day 28, or they withdraw or die. Twice a day, at approximately 11:00 and 15:00, study personnel perform the RASS, CAM-ICU, CAM-ICU-7, CPOT, and the Faces Anxiety Scale, evaluate mobility with the Hogdson Mobility Scale, and record adverse events.

Beginning within 72 h of extubation or removal of the endotracheal tube, and if the patient is CAM-ICU negative, the participant is reconsented for the study. The participant is also screened for obstructive sleep apnea with STOP-BANG and the Richards-Campbell Sleep Questionnaires, given the higher incidence of delirium in patients with sleep apnea and disturbed sleep quality [33–35].

### Follow-up

Participants are invited to follow-up in the Critical Care Recovery Center (CCRC) at 90 days from hospital discharge. The CCRC is an outpatient interdisciplinary clinic comprising care coordinators (social worker, nurse), a critical care physician, psychometrician, and psychologist. Its goal is to maximize the cognitive, physical, and psychological recovery of ICU survivors through comprehensive assessments and personalized recovery plans incorporating self-management protocols.

### Data collection

Prior to the trial initiation, study personnel underwent training sessions on data collection and were individually tested on data entry as well as outcome assessments.

Study data have been collected and managed using REDCap electronic data capture tools hosted at Indiana University. REDCap is a secure, web-based application designed to support data capture for research studies, providing: (1) an intuitive interface for validated data entry; (2) audit trails for tracking data manipulation and export procedures; (3) automated export procedures for seamless data downloads to common statistical packages; and (4) procedures for importing data from external sources.

On a daily basis during all phases of the study and for up to 28 days, blinded personnel collect vital signs, pertinent laboratory and imaging data, data to calculate severity of illness, clinical events including surgery and tracheostomy, duration and modes of mechanical ventilation, cumulative daily doses of sedatives, analgesics, antipsychotics, presence of shock requiring vasopressors, and the administration of systemic steroids or paralytic agents. We also collect disposition at discharge.

### Sample size and feasibility

We determined criteria for feasible recruitment rates based on prior research. A large trial of music in the ICU had a recruitment rate of 33%, while delirium studies have had recruitment rates close to 30% [11]. Our own recruitment experience at the institution where this trial is being conducted suggests recruitment rates near 60%. Given a sample size of 60 participants, we will be able estimate a recruitment rate of 60% to within a 95% confidence interval of ± 12.40%. A recruitment rate of 48% would be lower than our institutional norm, but remains higher than other similar trials.

Adherence to the intervention will be defined as the percentage of audio sessions completed per patient (a maximum of 14 sessions per patient at a rate of two sessions per day for 7 days), adjusted for number of eligible sessions based on length of stay in the ICU. Our sample size of 60 participants will permit an estimate of the adherence rate of 80% to within a 95% confidence interval of ± 10.12%. We determined a retention rate of 80% for feasibility, defined by the percentage of participants completing all in-hospital components of the study, which can be estimated with a similar confidence interval. As a pilot study, we anticipate that a sample size of 60 participants will be sufficient to determine feasibility.

Our pilot study will help calculate sample size needed for a definitive randomized controlled trial. A decrease of 15% in delirium incidence between groups would make a larger trial feasible, with a three-arm trial requiring 548 participants (allowing 20% for attrition, *α* = 0.05, power = 80%).

### Statistical analysis

We will use chi-square tests to determine if study retention rates differ across the three groups. Adherence will be defined as the percentage of audio sessions completed per patient, adjusted for number of eligible sessions based on length of stay in the ICU. Depending on the distribution of this outcome, we will use either analysis of variance (ANOVA) (for normally distributed data) or the Kruskal-Wallis test (non-normal data) to determine if adherence differs between the three study arms.

## Discussion

DDM is a feasibility trial for providing personalized and non-personalized music interventions for critically ill, mechanically ventilated patients. In our trial we also estimate the preliminary efficacy of music interventions on reducing delirium incidence and severity.

Our protocol builds on prior research as the anxiolytic properties of music have been well documented [12]. Music interventions for hospitalized and non-hospitalized patients have shown decreases in heart rate and blood pressure, and were hypothesized to be a consequence of a lower sympathetic drive [12, 14]. Earlier studies have also shown lower sedative doses for patients undergoing procedures and utilizing preferred music listening [11]. Prior studies have been limited by methodological challenges, including small sample size, lack of blinding, and exclusion of the critically ill. The strengths of our protocol include blinding of outcome assessors, twice daily delirium assessments, as well as assessments of pain, anxiety, and mobility milestones. Furthermore, our study benefits from high external validity through inclusion of the ICU’s sickest patients.

Music listening has been shown to activate areas of the brain involved with memory, cognitive function, and emotion [36, 37]. By reducing brain dysfunction and increasing activity in the areas related to memory, music could help retain cognitive function, particularly in older people who experience critical illness or injury. Extended applications of our study could permit comparison of neurocognitive outcomes between music and attention-control arms in future studies.

As a feasibility study, our protocol will enable us to perform hypothesis testing, develop music algorithms, and implement a music intervention protocol in a busy ICU. The results of this study will inform the protocol design of a larger study.

### Trial status

Enrollment is ongoing. Recruitment began in December 2016 and is expected to conclude in January 2018. Target enrollment for the study is 60 participants. The trial was retroactively registered at ClinicalTrials.gov.

## Additional file

Additional file 1: SPIRIT 2013 Checklist: recommended items to address in a clinical trial protocol and related documents. (DOC 122 kb)

## Abbreviations

CAM-ICU: Confusion Assessment Method for the Intensive Care Unit
DDM: Decreasing Delirium through Music
IQCODE: Information Questionnaire on Cognitive Decline in the Elderly
